# Physician Variability in Diastology Reporting in Patients With Preserved Ejection Fraction: A Single Center Experience

**DOI:** 10.7759/cureus.9062

**Published:** 2020-07-08

**Authors:** Raj Patel, Anjali R Desai, Puja Patel, Varun Vanka, Harshavardhan Ghadiam, Tinoy Kizhakekuttu

**Affiliations:** 1 Cardiology, University of Illinois College of Medicine at Peoria, Peoria, USA; 2 Internal Medicine, University of Illinois College of Medicine at Peoria, Peoria, USA; 3 Internal Medicine, American University of Antigua, Peoria, USA; 4 Internal Medicine, OSF St. Francis Medical Center/University of Illinois College of Medicine at Peoria, Peoria, USA

**Keywords:** echocardiography, diastolic heart failure, heart failure with preserved ejection fraction, diastolic dysfunction, quality improvement

## Abstract

To help standardize the assessment of diastolic dysfunction in the United States, the American Society of Echocardiography (ASE) released criteria for the assessment of diastology in patients with normal and abnormal ejection fraction. As heart failure with preserved ejection fraction (HFpEF) is a leading cause of morbidity and mortality in cardiac patients, it is imperative to assess diastology appropriately. Echocardiography is the mainstay in the assessment of diastolic function; with the new ASE guidelines, diagnosis is simplified especially in patients that have preserved baseline ejection fraction. Our study aimed to determine the extent of physician variability in diastology reporting at our medical center after the release of the new ASE criteria.

## Introduction

Congestive heart failure (CHF) is a leading cause of morbidity and mortality worldwide [1]. Heart failure can be divided, in general, into two sub-categories: heart failure with preserved ejection fraction (HFpEF or diastolic heart failure) and heart failure with reduced ejection fraction (HFrEF or systolic heart failure) [2]. Heart failure with normal/preserved systolic function can interchangeably be labeled diastolic dysfunction or diastolic heart failure [3].

Diastolic heart failure is defined as evidence of diastolic dysfunction via Doppler echocardiography or cardiac catheterization in the setting of preserved ejection fraction with clinical signs and symptoms consistent with CHF. Per recent American Society of Echocardiography (ASE) guidelines, preserved left ventricular ejection fraction is defined as EF between 52-74% (both men and women) [2].

Women are more prone to developing diastolic heart failure. Additionally, the major cause for diastolic heart failure includes uncontrolled/longstanding essential hypertension, generally occurring in up to 60% of patients with diastolic dysfunction [2]. Prior population-based studies have also identified hyperlipidemia, obesity, diabetes mellitus and atrial fibrillation as possible causes of diastolic dysfunction [4].

Doppler echocardiography has been the mainstay of diagnosis of diastolic dysfunction. Several echo findings/criteria have been identified to assist in the assessment of diastolic heart failure. Due to lack of consensus on diastology reporting, in 2016, the American Society of Echocardiography released a standardized algorithm for the diagnosis of heart diastolic dysfunction in patients with normal ejection fraction [2]. These criteria include:

Septal e’<7 cm/sec or lateral e’ <10 cm/sec

Average E/e’ >14

Left atrial volume index >34 mL/m2

Peak tricuspid regurgitation velocity >2.8 m/sec

Using the above criteria in patients with preserved ejection fraction, diastolic dysfunction is present if >50% of the criteria are met (at least three positive), indeterminate if two criteria are met, and not present if <50% (one or none positive) criteria is met.

Our study aimed to determine the physician variability in diastology reporting at our medical center.

## Materials and methods

We retrospectively analyzed transthoracic echocardiograms performed from December 2017 to April 2018. Patients with an ejection fraction of 55% or more were included in our study. Transthoracic echocardiograms were evaluated and individually assessed for diastolic function based on the above guidelines and compared to physician reported diastology. All statistical analysis was done using R version 3.4.4 and with a two-sided confidence level of 95%. Data was provided for 831 patients from December 1st, 2017 to April 1st, 2018. Diastolic function was considered to be properly assessed when there was agreement between the physician summary and diastolic function grading based on the new ASE guidelines. Ninety-two patients were excluded due to an incomplete echocardiographic assessment with a total of 738 patients remaining in our cohort.

## Results

Agreement between the echo summaries and diastology on the initial three levels (yes, no and indeterminate) categorical variable was 57.6%, meaning the echo summaries did not match the diastology results 42.4% of the time. When the echo summary and diastology variables were transformed from a category with three levels to binary variables, indicating whether or not there was a positive diagnosis of diastolic dysfunction, the accuracy rate of the echo summaries was 78.2%, meaning they were correct 78.2% of the time but incorrect 21.8% of the time. The predictive performance of the echo summaries was calculated using the diastology as the “gold standard” for the diagnosis of diastolic dysfunction. A McNemar’s chi-square test found a significant difference in the proportion of patients with positive diastology, 10.03%, compared to the proportion of patients classified as positive for diastolic dysfunction by the echo summaries, 24.00%, c2 (1, N = 738) = 65.9, p < 0.001. The echo summaries had a sensitivity of 0.608, meaning that they correctly identified 60.8% of the patients with a positive diastology as positive for diastolic dysfunction, and a specificity of 0.80, meaning they correctly identified 80% of the patients with a negative diastology as negative for diastolic dysfunction.

**Figure 1:**
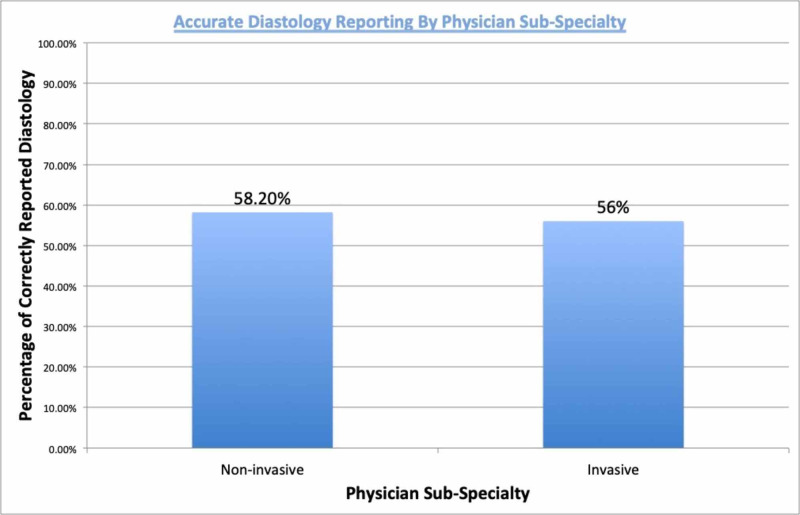
Accurate diastology reporting by physician sub-specialty

A total of 17 physicians were included in the study. A chi-square test did not find a significant difference in the proportion of proper assessments between non-invasive physicians (58.24%) and invasive physicians (56.00%), c2 (1, N = 733) = 0.27 (Figure 1).

**Figure 2:**
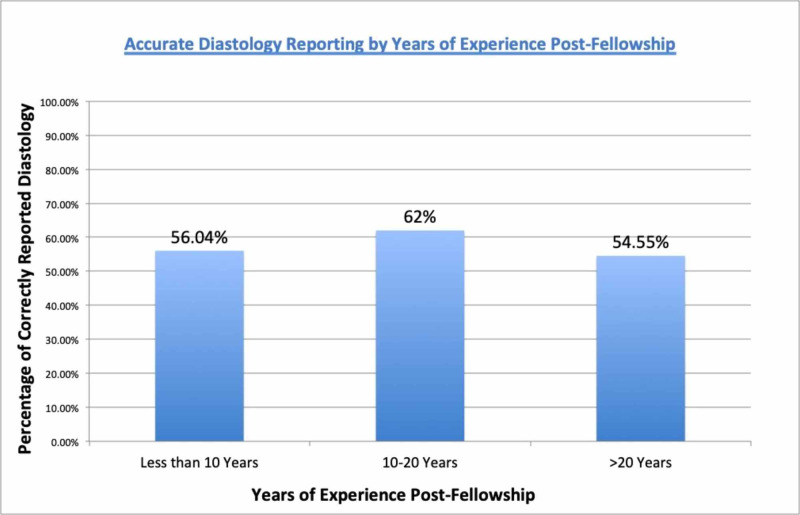
Accurate diastology reporting by years of post-fellowship experience

The 17 total physicians were grouped by years of experience, with five having 10 or less years of experience, four having 11 to 20 years of experience and eight having 20 or more years of experience. A total of 298 echo studies were read by the 10 or less years of experience group, 237 echos were read by the 11 to 20 years groups and 198 studies were read by the group with 20 or more years of experience. Faculty with less than 10 years post-fellowship experience reported diastology properly 56.04% of the time; 11-20 years of post-fellowship practice reported diastology properly 62% of the time, and faculty with more than 20 years of experience reported diastology appropriately 54.55% of the time (Figure 2).

No significant differences were found with the pairwise comparisons or an overall 2 x 3 chi-square test, c2 (2, N = 733) = 3.33, p = 0.19.

## Discussion

Diastolic heart failure is a common disease process that affects nearly 5 million patients in the United States with 500,000 new cases diagnosed yearly. Approximately 50% of all patients diagnosed with heart failure have diastolic heart failure and it is a leading cause of morbidity and mortality. Described generally as decreased compliance and impaired relaxation due to a stiffened left ventricle, diastolic heart failure mainly afflicts elderly, hypertensive females. However, any condition that may alter the compliance or relaxation of the left ventricle may lead to diastolic dysfunction and diastolic heart failure. Ischemia, atrial fibrillation, and general aging have been reported as major contributors to developing diastolic dysfunction [5].

Diastolic dysfunction and diastolic heart failure are not interchangeable terms. Whereas diastolic dysfunction is a preclinical state that generally has echocardiographic and invasive hemodynamic abnormalities, diastolic heart failure is the presence of dysfunction in the setting of heart failure symptoms. Diastolic heart failure or heart failure with preserved ejection fraction has identical clinical findings and symptoms with systolic heart failure. Additionally, the neuro-hormonal abnormalities, decrease in exercise capacity, and reduced exercise-induced cardiac output generally seen with systolic heart failure are also seen in diastolic heart failure [6].

Left ventricular fibrosis has been a postulated mechanism of diastolic dysfunction. Long-standing hypertension, disease processes such as ischemia or restrictive cardiomyopathies may cause myocardial death and resultant fibrosis. Therapies to reduce ventricular fibrosis have shown to reduce the progression of diastolic dysfunction [7]. At the current juncture, the mainstay of therapy is control of hypertension and maintaining euvolemia with diuretic therapy in addition to treating an underlying/reversible cause of diastolic dysfunction [8]. Angiotensin-converting enzyme (ACE) inhibitors, angiotensin receptor blockers (ARBs), aldosterone antagonists, beta blockers, and calcium channel blockers have shown to be beneficial in patients with diastolic dysfunction [9].

Since the advent of echocardiographic assessment of diastolic function approximately 30 years ago, guidelines have continued to evolve and mature in an attempt to assist imagers/interpreters in the accurate assessment of diastology [10]. However, despite multiple guidelines and criteria for the assessment of diastolic function, there seemed to be continued physician variability in report diastology. Thus, in 2016, the American Society of Echocardiography/European Association of Cardiovascular Imaging released simplified guidelines for the assessment of diastolic function in patients with preserved/normal left ventricular ejection fraction [2].

Despite these newly recommended guidelines for assessment of diastolic function, we hypothesized that there continues to be physician variability in our clinical center. This was confirmed with our results as only 57.6% of physicians reported diastolic function accurately based on what was reported in the echo summary compared to diastolic function measured by the new ASE/ESCI guidelines. There was no significant difference in reporting between interventional/invasive faculties compared to non-invasive faculty. Additionally, years of practice beyond fellowship was not found to be statistically significant for accurate assessment of diastolic function. This data is important as it provides the foundation for continued improvement throughout all specialties and experience levels.

We performed a thorough search of prior studies and review articles; no prior studies have been done to assess the accuracy of physician reporting of diastolic function. We hope our study can help the impetus for inter-facility quality improvement in echocardiography assessment.

Given the significant morbidity, mortality, and healthcare costs associated with patients diagnosed with diastolic dysfunction/diastolic heart failure, continued education of physicians, fellows in training, and echocardiography staff should be undertaken to enhance and universalize the skills/knowledge needed to accurately assess diastolic function.

## Conclusions

Diastolic dysfunction and diastolic heart failure are common diagnoses and leading causes for morbidity and mortality in cardiac patients. As echocardiography has become the mainstay of diagnosis, it is imperative that echo readers are familiar with the latest guideline recommendations on the assessment of diastolic dysfunction. Our study highlights that there is still room for improvement in diastology reporting by physicians irrespective of years in practice or sub-specialization. We hope our study can promote more awareness and guide future studies on quality improvement in diastology diagnosis and reporting.
